# New Properties of Drosophila scs and scs’ Insulators

**DOI:** 10.1371/journal.pone.0062690

**Published:** 2013-04-24

**Authors:** Olga Kyrchanova, Dmitry Leman, Alexander Parshikov, Anna Fedotova, Vasily Studitsky, Oksana Maksimenko, Pavel Georgiev

**Affiliations:** 1 Group of Transcriptional Regulation, Institute of Gene Biology, Russian Academy of Sciences, Moscow, Russia; 2 Department of the Control of Genetic Processes, Institute of Gene Biology, Russian Academy of Sciences, Moscow, Russia; 3 School of Biology, Moscow State University, Moscow, Russia; 4 Department of Pharmacology, UMDNJ–Robert Wood Johnson Medical School, Piscataway, New Jersey, United States of America; Université Paris-Diderot, France

## Abstract

Insulators are defined as a class of regulatory elements that delimit independent transcriptional domains within eukaryotic genomes. The first insulators to be identified were scs and scs', which flank the domain including two *heat shock 70* genes. Zw5 and BEAF bind to scs and scs', respectively, and are responsible for the interaction between these insulators. Using the regulatory regions of *yellow* and *white* reporter genes, we have found that the interaction between scs and scs' improves the enhancer-blocking activity of the weak scs' insulator. The sequences of scs and scs' insulators include the promoters of genes that are strongly active in S2 cells but not in the eyes, in which the enhancer-blocking activity of these insulators has been extensively examined. Only the promoter of the *Cad87A* gene located at the end of the scs insulator drives *white* expression in the eyes, and the *white* enhancer can slightly stimulate this promoter. The scs insulator contains polyadenylation signals that may be important for preventing transcription through the insulator. As shown previously, scs and scs' can insulate transcription of the *white* transgene from the enhancing effects of the surrounding genome, a phenomenon known as the chromosomal position effect (CPE). After analyzing many independent transgenic lines, we have concluded that transgenes carrying the scs insulator are rarely inserted into genomic regions that stimulate the *white* reporter expression in the eyes.

## Introduction

Enhancer-mediated activation is a fundamental mechanism of gene regulation in eukaryotes [1], [2]. Enhancers interact with tagged genes by looping out the intervening sequences. The putative ability of enhancers to stimulate unrelated promoters has provided a basis for the model suggesting the existence of a specific class of regulatory elements, named insulators, that form independent transcriptional domains and preclude undesirable interactions between enhancers and promoters [3]–[10]. Insulators have two properties: (1) they prevent enhancers and silencers from communicating with a promoter only when inserted between such regulatory elements and a promoter [11]–[16] and (2) protect gene expression from positive and negative chromatin position effects [17]–[19].

The second property of insulators has been mainly examined using the *white* reporter in transgenic *Drosophila* lines [17], [19]–[23]. Flies carrying the *white* transgene without the upstream regulatory region (*mini-white*) display a wide variety of eye colors depending on the transgene insertion site, a phenomenon referred to as the chromosomal position effect (CPE) [24], [25]. To explain the high sensitivity of the *mini-white* gene to chromosomal position effects, it has been suggested that the *white* promoter can function as an enhancer trap, meaning that enhancers located either 5' or 3' of the transposon are able to stimulate transcription of the *mini-white* gene. However, we have recently found that, in more than 70% of cases, transcription through the *mini-white* gene is responsible for positive position effects [26]. Consistently with this finding, transcriptional terminators proved to be efficient in protecting *mini-white* expression from CPE.

**Figure 1:**
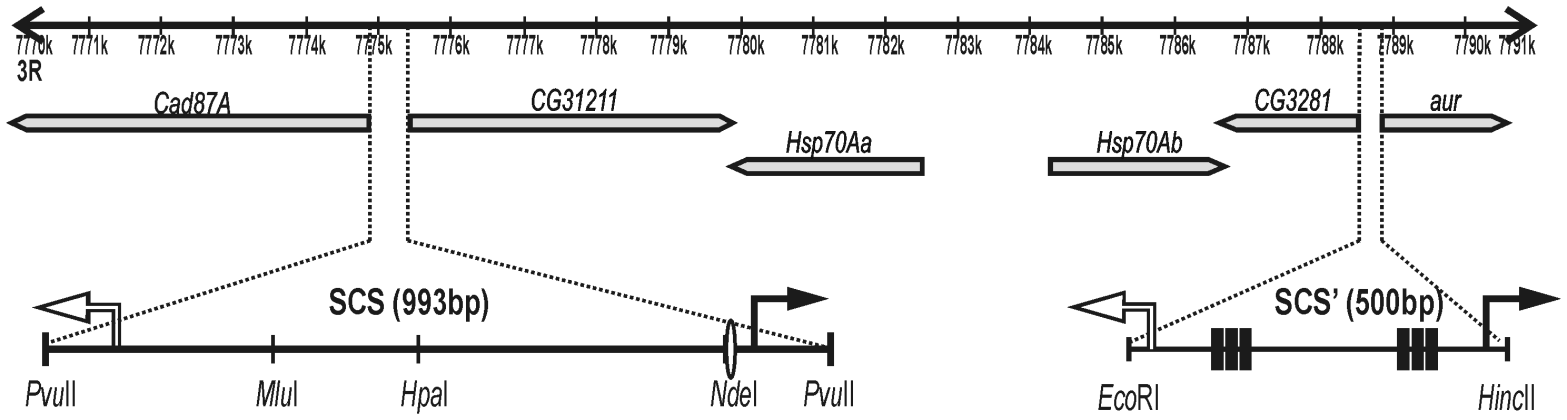
Genomic region containing the ***hsp70*** **genes (FlyBase data).** This 15-kb region contains six genes (shown as gray arrows): a pair of divergently transcribed *hsp70* genes, *Cad87A, CG31211, CG3281*, and *aurora.* Dotted lines show locations of scs (993 bp) and scs' (500 bp). Black arrows indicate positions of the *aur* and *CG31211* promoters. White arrows indicate positions of the *Cad87A* and *CG3281* promoters. The Zw5 binding site within scs is shown as a white oval. Positions of BEAF binding sites within scs' are shown as black rectangles.

The first *Drosophila* insulators to be identified were scs and scs', which flank the 14-kb region containing five genes (Figure 1), including two *heat shock 70* genes [17], [27], [28]. It has been shown that the scs and scs' insulators protect from CPE [17], [21] and that multiple sequences within scs and scs' are required for their insulator function [29]–[31]. Two proteins, Zw5 and BEAF, bind to and partially confer the insulator function to scs and scs', respectively [30]–[32]. According to the chromosome conformation capture assay, scs and scs' pair with each other *in vivo* [33]. The Zw5 and BEAF proteins interact *in vitro* and *in* *vivo,* which is evidence for their involvement in the formation of a chromatin loop between the scs and scs' insulators [33]. However, the role of such a chromatin loop in forming an independent chromatin domain has not been demonstrated.

In contrast to classic insulators, scs and scs' are not neutral chromatin domain boundaries [34] but contain promoter regions that may be involved in the enhancer-blocking activity of these insulators. The scs' insulator sequence (approximately 500 bp) includes the promoters of the *CG3281* and *aurora* genes (FlyBase database). In the scs insulator, the *CG31211* and the *Cad87A* promoters are located at the ends of its 993-bp sequence [35, FlyBase database]. Recent genome-wide studies have identified binding sites for BEAF and Zw5 proteins as preferentially located in promoter regions [36]–[39]. These and other recent data suggest that insulators might have evolved as specialized derivatives of promoters and that the two types of elements employ related mechanisms to mediate their distinct functions [8], [40]. However, functionality of these promoters and their contribution to the activities of scs and scs' insulators have not been examined.

Since characterization of BEAF and ZW5 as insulator proteins is impossible without the results obtained with the scs and scs' insulators themselves, we examined the properties of these insulators in transgenic lines using the model of *yellow* and *white* regulatory regions. As a result, we found that scs improves the enhancer-blocking activity of scs', supporting the functional interaction between these insulators. According to an assay in *Drosophila* S2 cells, both scs and scs' contain functional promoters at their ends, but only the *Cad87A* promoter of scs can effectively drive *white* transcription in the eyes. The scs insulator contains terminators that may be important for preventing transcription through the insulator. In addition, it decreases the frequency of integration of the *mini-white* transgene into genes actively transcribed in the eyes. This may shed some light on the mechanism of scs-mediated blocking of the chromosomal position effects.

## Materials and Methods

### Generation and Analysis of Transgenic Lines

The study was performed with 993-bp scs, 500-bp scs', zw5^×4^, zw5^×8^, and 852-bp Wary fragments, which were obtained as described [41], [42] and cloned between lox or frt sites. The constructs were based on the CaSpeR vector [43]. The Wari insulator located on the 3′ side of the *mini-white* gene was deleted from CaSpeR to produce plasmid pCaSpeRΔ700. The *Eco*RI restriction site was inserted at 3' *mini-white* end for cloning the test elements in some constructs. The constructs with *yellow* and *white* reporter genes for testing enhancer-blocking activity was made as described previously. The test insulator fragments were cloned at –893 relative to the *yellow* transcription start site. Details of plasmid construction and their schemes are available upon request.

The construct and P25.7 wc plasmid were injected into *yacw^1118^* preblastoderm embryos [44]. The resultant flies were crossed with *yacw^1118^* flies, and the transgenic progeny were identified by their eye color. The lines with DNA fragment excisions were obtained by crossing transposon-bearing flies with the Flp (*w^1118^; S2CyO, hsFLP, ISA/Sco;+*) or Cre (*yw; Cyo, P[w+,cre]/Sco;+*) recombinase-expressing lines. The Cre recombinase induces 100% excisions in the next generation [45]. A high level of Flp recombinase was produced by heat shock treatment for 2 h during the first 3 days after hatching [46]. All excisions were confirmed by PCR analysis. Details of the crosses and primers used for genetic analysis and excision of functional elements are available upon request.

To induce GAL4 expression, we used the modified *yw^1118^; P[w^–^, tubGAL4]117/TM3,Sb* line (Bloomington Center #5138) in which the marker *mini-white* gene was deleted as described [47].

To estimate the levels of *yellow* and *white* expression, we visually determined the degree of pigmentation in the abdominal cuticle and wing blades (*yellow*) and in the eyes (*white*) of 3- to 5-day-old males developing at 25°C, with reference to standard color scales. In the five-grade scale for *yellow*, grade 5 corresponds to wild type, and grade 1, to the total loss of *yellow* expression. Identical data were obtained for the wing and body pigmentation in all experiments. In the nine-grade scale for *white*, brick red (R) eyes correspond to wild type, and white eyes (W), to the total loss of *white* expression. Intermediate levels of eye pigmentation, in order of decreasing gene expression, are brownish red (BrR), brown (Br), dark orange (dOr), orange (Or), dark yellow (dY), yellow (Y) and pale yellow (pY).

Two experts separately inspected 30–50 flies from each of two independent crosses for every transgenic line. Each line thus assessed contributed a unit to the corresponding cell of the scoring table. Hence, each numerical entry in the distributions shown in the figures under the scales is the number of fly lines with the specified pigmentation grade (corresponding to the gene expression level decreasing from left to right).

Construct insertion sites in transgenic lines were determined with inverse PCR technique. Genomic DNA extracted from transgenic flies was treated with *Rsa*I or *Mbo*I endonuclease. The cleaved DNA was ligated and PCR-amplified with primers 5′-aagattcgcagtggaaggctgcac-3′and 5′-tccgcacacaacctttcctctcaac-3′ (after *Rsa*I cleavage) or 5′-cccttagcatgtccgtggggtttg-3′ and 5′-cgctgtctcactcagactcaatacgacac-3′ (after *Mbo*I cleavage). The PCR products were sequenced, and the coordinates and directions of insertions were determined with the Flybase R5.13 database.

### Construction of Plasmid Reporter System and Dual Luciferase Assay

Constructs for promoter and terminator assays were generated in pAc5.1/V5-His B (Invitrogen). The *firefly* and *Renilla* luciferase sequences were taken from pGL3Basic and pRL-CMV vectors (Promega), respectively. In the control plasmid, the firefly luciferase ORF without the promoter sequence was used. Potential promoter elements were inserted upstream of the *firefly* ORF. To normalize the firefly data, the promoter assay was performed with the plasmid containing the *Renilla* luciferase ORF under actin promoter. For terminator assay, we generated a bicistronic system with *Renilla* and firefly luciferases sequentially located downstream of the general actin promoter. For the basic construct, the *reaper* gene IRES was amplified from genomic DNA and cloned between the luciferase sequences. The SV40 terminator sequence was taken from pAc5.1/V5-His B vector. SV40 terminator and scs insulator were inserted upstream of IRES.

*Drosophila* Schneider 2 cells were grown in SFX medium (HyClone) at 25°C. Their transfection with plasmids was performed using the Cellfectin II reagent (Invitrogen) according to the manufacturer's instructions, in six-well plates at a density of 10^6^ cells/ml, with the cells being grown for 24–48 hours before harvesting. The firefly luciferase data were normalized relative to the *Renilla* luciferase data. The dual luciferase assay was performed with the Firefly & Renilla Luciferase Assay Kit (Biotium). At least three independent experiments were performed for three independent transfection procedures.

## Results

### Testing the scs and scs' Insulators for Enhancer Blocking Activity

**Figure 2:**
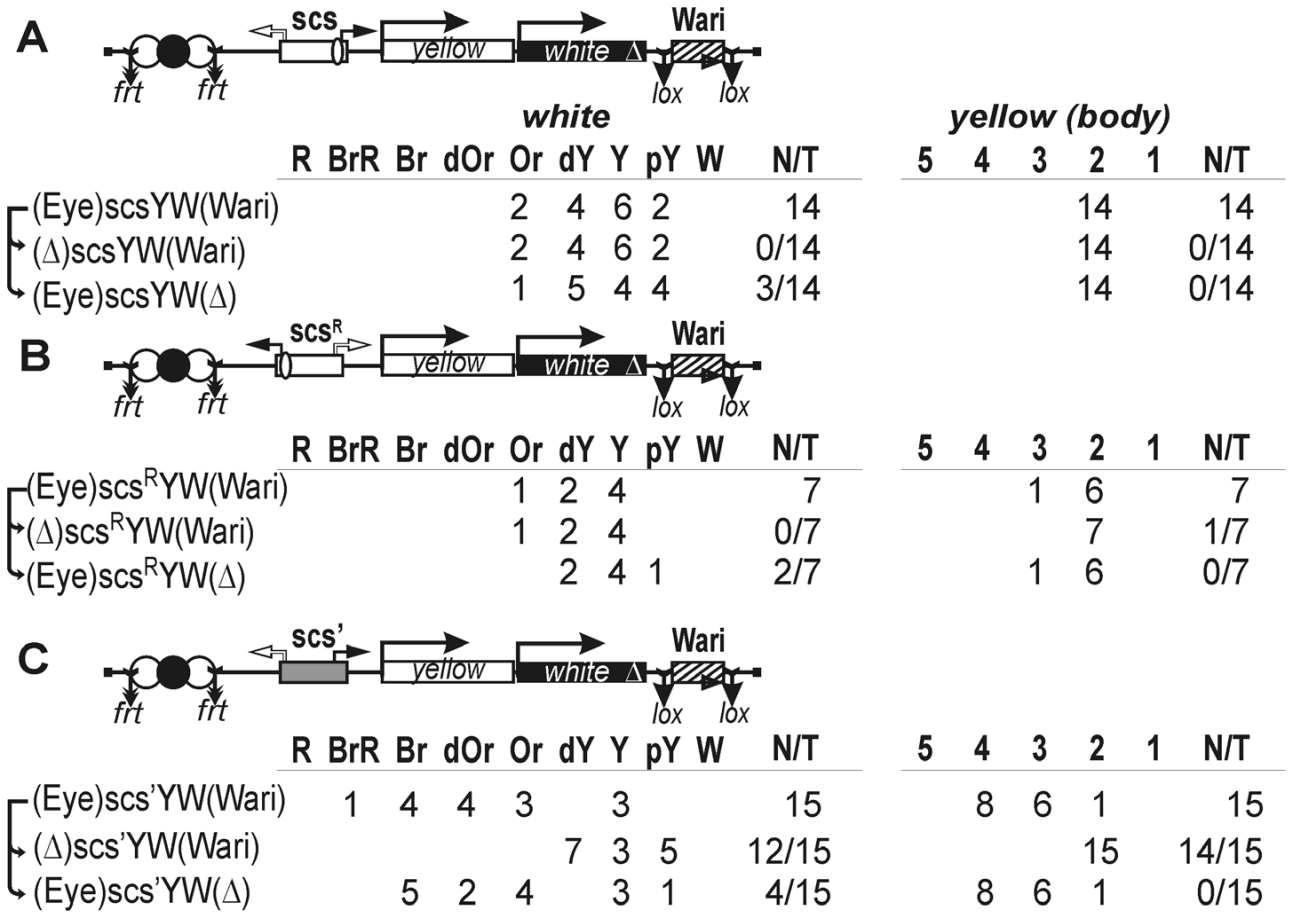
The role of the Wari insulator in the enhancer-blocking activity of scs and scs' insulators in transgenic lines. Tests were conducted for the functional interaction of Wari with the scs insulator inserted in (A) direct or (B) reverse orientation and (C) with the scs' insulator. In the reductive scheme of the transgenic construct used in the assay, the *white* and *yellow* genes are shown as white and black boxes, respectively, with an arrow indicating the direction of transcription; the delta sign (Δ) indicates deletion of Wari located at the 3' end of the *white* gene; downward arrows indicate target sites for Flp recombinase (*frt*) or Cre recombinase (*lox*); the same sites in construct names are denoted by parentheses; the eye enhancer is shown as black oval; the *yellow* wing and body enhancers are shown as white ovals. The “*white*” column shows the number of transgenic lines with different levels of eye pigmentation. Arrows indicate the excision of an element to produce the derivative transgenic lines. Wild-type *white* expression determined the bright red eye color (R); in the absence of *white* expression, the eyes were white (W). Intermediate levels of pigmentation, with the eye color ranging from pale yellow (pY), through yellow (Y), dark yellow (dY), orange (Or), dark orange (dOr), and brown (Br) to brownish red (BrR), reflect the increasing levels of *white* expression. The “*yellow*” column shows the numbers of transgenic lines with the *yellow* pigmentation level in the abdominal cuticle (reflecting the activity of the body enhancer); in most of the lines, the pigmentation level in wing blades (reflecting the activity of the wing enhancer) closely correlated with these scores. The level of pigmentation (i.e., of *y* expression) was estimated on an arbitrary five-grade scale, with wild-type expression and the absence of expression assigned scores 5 and 1, respectively. N is the number of lines in which flies acquired a new white or yellow phenotype after deletion (Δ) of the specified DNA fragment; T is the total number of lines examined for each particular construct. Other designations are as in Figure 1.

The scs and scs' insulators were previously tested in the transgenes carrying the *mini-white* gene as a reporter or selection marker [13], [28], [29], [48]–[51]. We found that the endogenous insulator, named Wari, was located at the 3' end of the endogenous *white* gene and the *mini-white* gene used in the constructs [41] and showed that Wari improved the enhancer-blocking activity of Su(Hw)-dependent insulators. To find out whether the Wari insulator is required for the enhancer-blocking activity of scs and scs' insulators, we used the previously described model system with two reporter genes, *yellow* and *white* (Figure 2). The y*ellow* gene accounts for dark pigmentation of larval and adult cuticle and its derivatives, with two upstream enhancers being responsible for its activation in the body cuticle and wing blades [52]. The *white* gene is responsible for eye pigmentation, and its expression in the eyes is activated by a specific enhancer [53]. In our experiments, the eye enhancer was inserted between the wing and body enhancers (collectively designated as Eye, Figure 2). All enhancers flanked by frt sites were inserted in front of the *yellow* gene. The *white* gene was inserted on the 3' side of the *yellow* gene. In this configuration, the eye enhancer–*white* promoter communication was partially attenuated by the *yellow* promoter (data not shown). The endogenous Wari insulator was deleted from the constructs, flanked by lox sites, and reinserted at the same place.

The 993-bp scs insulator was inserted in either direct (Figure 2A) or reverse orientation (Figure 2B) between the enhancers and the *yellow* promoter. In all transgenic lines, flies had yellow pigmentation of wing blades and body cuticle, and eye pigmentation ranged from pale yellow to orange, indicating that the enhancer were unable to activate the reporter genes. This conclusion was supported by the fact that deletion of the enhancers resulted in only a slight decrease in *yellow* and *white* expression. Deletion of the Wari insulator led to reduction of eye pigmentation in five transgenic lines but did not affect *yellow* expression (Figures 2A, 2B). In the light of our previous observations [54], we consider that the slight positive effect of the Wari insulator results from a positive influence on the *white* promoter rather than from an interaction with scs. Taken together, these results show that the Wari insulator is not required for the enhancer-blocking activity of the strong scs insulator.

Next, we inserted the scs' insulator between the enhancers and the *yellow* promoter (Figure 2C). In transgenic lines, flies had a moderate level of wing and body pigmentation, suggesting partial activation of the *yellow* promoter by the enhancers. Likewise, transgenic flies had the eye color ranging from yellow to brown-red, which was indicative of *white* stimulation by the eye enhancer in some transgenic lines. Indeed, deletion of the enhancers proved to considerably reduce the *yellow* and *white* expression. Thus, the results of these experiments confirmed previous observations that scs' is a relatively weak insulator [48], [49]. Once again, deletion of the Wari insulator did not affect the enhancer-blocking activity of the scs’ insulator. Taken together, these results provide evidence that the scs and scs' insulators do not functionally interact with the Wari insulator.

### Testing for the Functional Interaction between the scs and scs' Insulators

**Figure 3:**
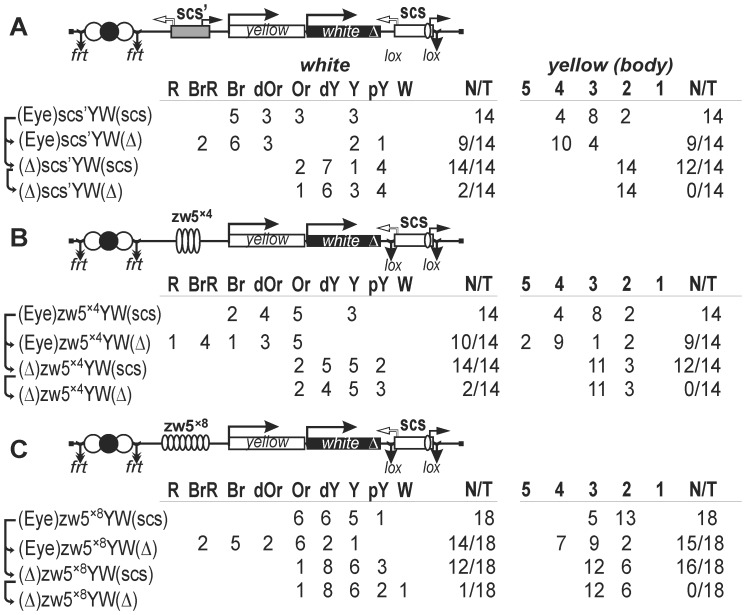
Testing for the functional interaction between (A) scs' or (B–C) Zw5 binding sites and the scs insulator located on the 3' side of the *white* gene. Other designations are as in Figures 1 and 2.

There is evidence that scs and scs' interact *in vivo* [33], but the functional role of their interaction has not been demonstrated. Therefore, we then used the same transgenic assay with the *yellow* and *white* genes to find out if a chromatin loop formed by the scs and scs' insulators could improve enhancer blocking. Since a single copy of scs completely blocked the *yellow* and *white* enhancers, we inserted the weak scs' insulator between the enhancers and the *yellow* gene (Figure 3A). The scs insulator flanked by lox sites was inserted instead of the Wari insulator downstream of the *white* gene (Figure 3A). As a result, the scs and scs' insulators formed a 9226-bp chromatin domain including two reporter genes, which corresponded to the distance between these insulators in their endogenous positions at the ends of the domain containing the *heat shock 70* genes [27].

In 14 transgenic lines, y and w phenotypes of flies indicated that the enhancers were partially active (Figure 3A). Deletion of the scs insulator resulted in an enhancement of eye pigmentation in seven transgenic lines and of wing and body pigmentation in nine transgenic lines. Thus, scs partially improved the enhancer-blocking activity of the scs' insulator.

The scs insulator contains a binding site for the Zw5 protein, which is required for the enhancer-blocking activity [31]. It was shown that four Zw5 binding sites partially blocked the eye enhancer, and we previously found that Zw5 binding sites supported distant interactions between regulatory elements in transgenic lines [42]. To test if scs can improve the enhancer-blocking activity of Zw5 binding sites, we inserted either four (Figure 3B) or eight such sites (Figure 3C) between the enhancers and the *yellow* promoter. The enhancer-blocking activity proved to be stronger in transgenic lines with eight, rather than four, Zw5 binding sites. In both cases, deletion of the scs insulator considerably improved *yellow* and *white* expression, suggesting that the scs insulator functionally interact with the Zw5-binding regions in blocking the enhancer activity.

### Testing for the Promoter and Transcription Terminator Activity of scs and scs' Insulators in S2 Cells

**Figure 4:**
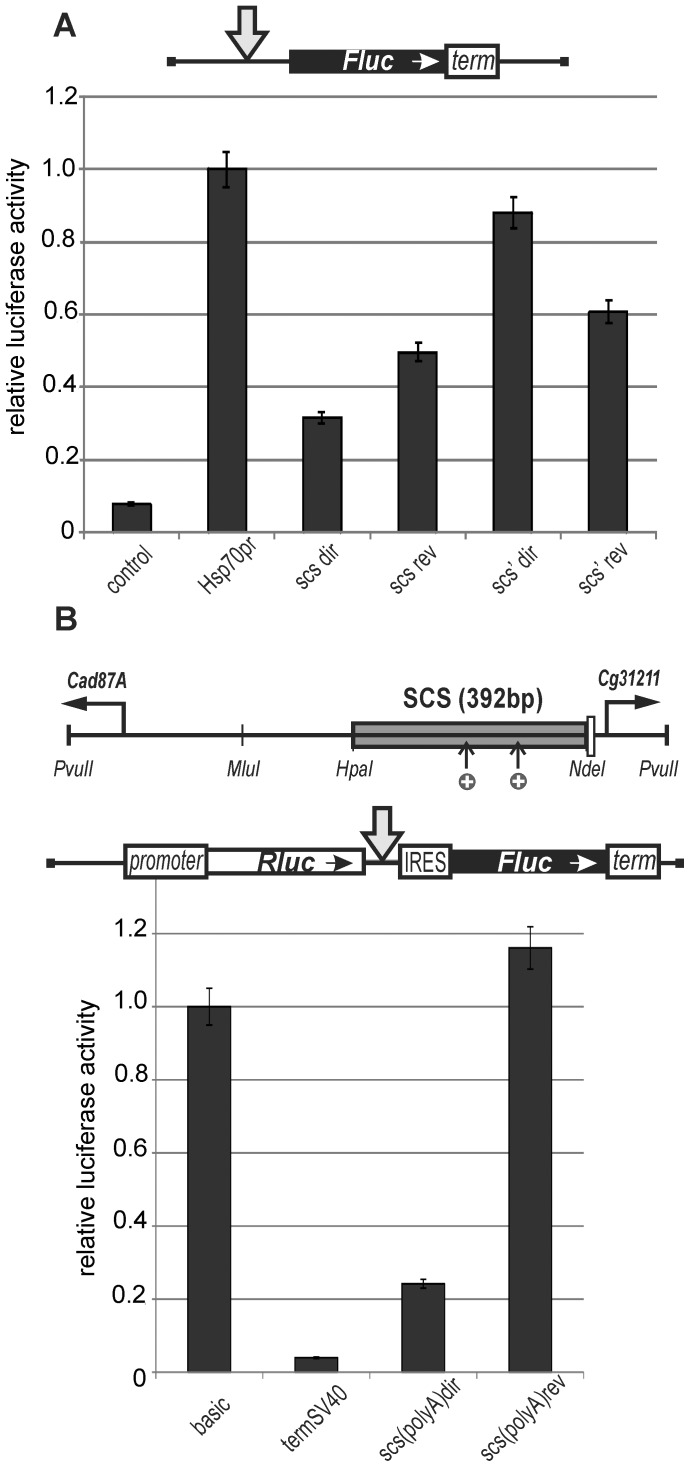
Testing elements for (A) promoter and (B) terminator activities in S2 cells. In the structural scheme of the scs insulator (*Pvu*II–*Pvu*II fragment), the Zw5 site is shown as a white rectangle; identified polyadenylation sites (PAS), as black circles with “+” sign indicating their direct orientation. Restriction sites *Hpa*I–*Nde*I indicate the boundaries of the element used in the terminator assay. In the reductive schemes of transgenic constructs, the ORFs encoding *Renilla* (*Rluc*) and firefly (*Fluc*) luciferases are shown as white and black boxes with arrows indicating the direction of transcription; the white rectangle marked “term” is the SV40 terminator. The bicistronic plasmid also contained the *actin* promoter and *rpr* IRES (rectangles with corresponding marks). Thick downward arrows indicate insertion sites for the *hsp*70 promoter (Hsp70 pr), scs (scs dir, scs rev), and scs' (scs' dir, scs' rev) in the promoter assay and for late SV40 (termSV40) and scs PASs (scs(polyA)dir, scs(polyA)rev) in the terminator assay. The reporter system used in the assays is based on measurement of Fluc versus Rluc activity. The Fluc/Rluc ratios for the test constructs are shown in histograms. Error bars show standard deviations (*n* = 3).

Previously, promoters were mapped at the ends of the scs' and scs sequences [34], [35], [49] (Figure 1). To check if the scs and scs' insulators used in the studies included all sequences necessary for promoter activity, we tested them for this activity in S2 cells using a luciferase reporter assay. As a result, we found that both ends of the scs and scs' insulators contained functional promoters that could drive luciferase transcription at a level comparable to that of the *hsp70* promoter (Figure 4A).

According to sequence data, the central part of scs contains two polyadenylation signals that match potential transcription terminators operating in direct orientation (Figure 4B). To test for transcription terminator activity in the scs insulator, we used a bicistronic reporter based on two luciferase coding sequences driven by a single *Drosophila actin 5C* promoter. The IRES sequence from the *Drosophila reaper* gene [55] was inserted between *Renilla* luciferase (*Rluc*) and firefly luciferase (*Fluc*) (Figure 4B).

It was expected that if poly(A) signal was functional, a monocistronic *Rluc* mRNA would be produced; if poly(A) signal was non-functional or weakly functional, a longer mRNA would be generated, reaching the SV40 poly(A) signal located downstream of the *Fluc*. Thus, the Fluc-to-Rluc ratio would allow us to estimate the amount of long bicistronic mRNA relative to the total mRNA transcribed from construct.

In the bicistronic reporter, we inserted either SV40 terminator as a control or the central 392-bp *Hpa*I–*Nde*I part of scs (scs^m^) that contains two polyadenylation signals (Figure 4B). As a result, we observed that the test scs fragment had a strong transcription terminator activity only in the direct orientation, corresponding to the presence of two polyadenylation signals. Thus, the scs insulator can function as a transcription terminator.

### Testing the scs Insulator in the Promoterless *white* Assay

Our results suggested that the scs insulator contained two functional promoters at the ends and terminators in the middle.

**Figure 5:**
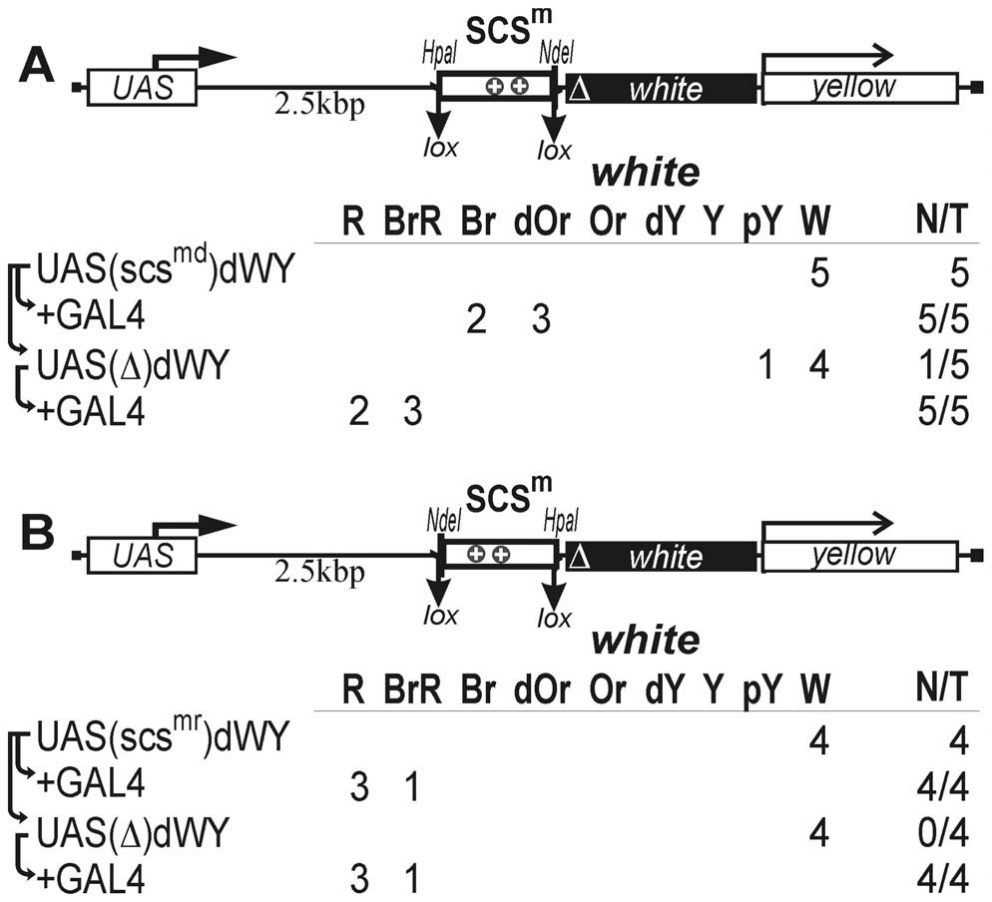
Testing the central 392-bp *Hpa*I–*Nde*I part of scs (scs^m^) inserted in either (A) direct or (B) reverse orientation for the ability to terminate transcription in the eyes. The UAS promoter is shown as the white rectangle marked “UAS.” “+GAL4” indicates that eye phenotypes in transgenic lines were examined after induction of GAL4 expression. In this case, N is the number of lines in which flies acquired a new w phenotype upon induction of GAL4. For other designations, see Figures 1 and 2.

To test the terminators in scs for the ability to arrest transcription elongation in the eyes, we used a model system that contained the UAS promoter, a 2-kb spacer from the *lacZ* gene, and the promoterless *mini-white* gene with deleted Wari insulator (Figure 5). The *white* gene also contains an internal ribosome entry site that helps to translate mRNAs from the internal sites [26]. The *yellow* gene was used as a marker for selecting transgenic lines. The central 392-bp *Hpa*I–*Nde*I part of scs (scs^m^) containing two polyadenylation signals flanked by lox sites was inserted into the spacer in either the direct (Figure 5A) or reverse orientation (Figure 5B). To express the GAL4 protein, we used the transgenic line carrying the GAL4 gene under control of the ubiquitous *tubulin* promoter (*tubGAL4*). The transgenic flies carrying the fragment of scs in either orientation had white eyes. Upon induction of the UAS promoter by crossing with the *tubGAL4* line, flies carrying the scs fragment in direct orientation acquired eye pigmentation from dark orange to brown (Figure 5A). In derivative transgenic lines obtained by deleting the scs fragment, induction of the UAS promoter by GAL4 expression resulted in eye pigmentation ranging from brown-red to red, which was indicative of strong *mini-white* activation in transgenic flies. In contrast, GAL4 stimulated *mini-white* expression to the same level (brown-red eye pigmentation) in the presence or absence of the scs fragment inserted in reverse orientation (Figure 5B). These results suggest that the terminators contained in scs are functional in the transgenic lines.

**Figure 6:**
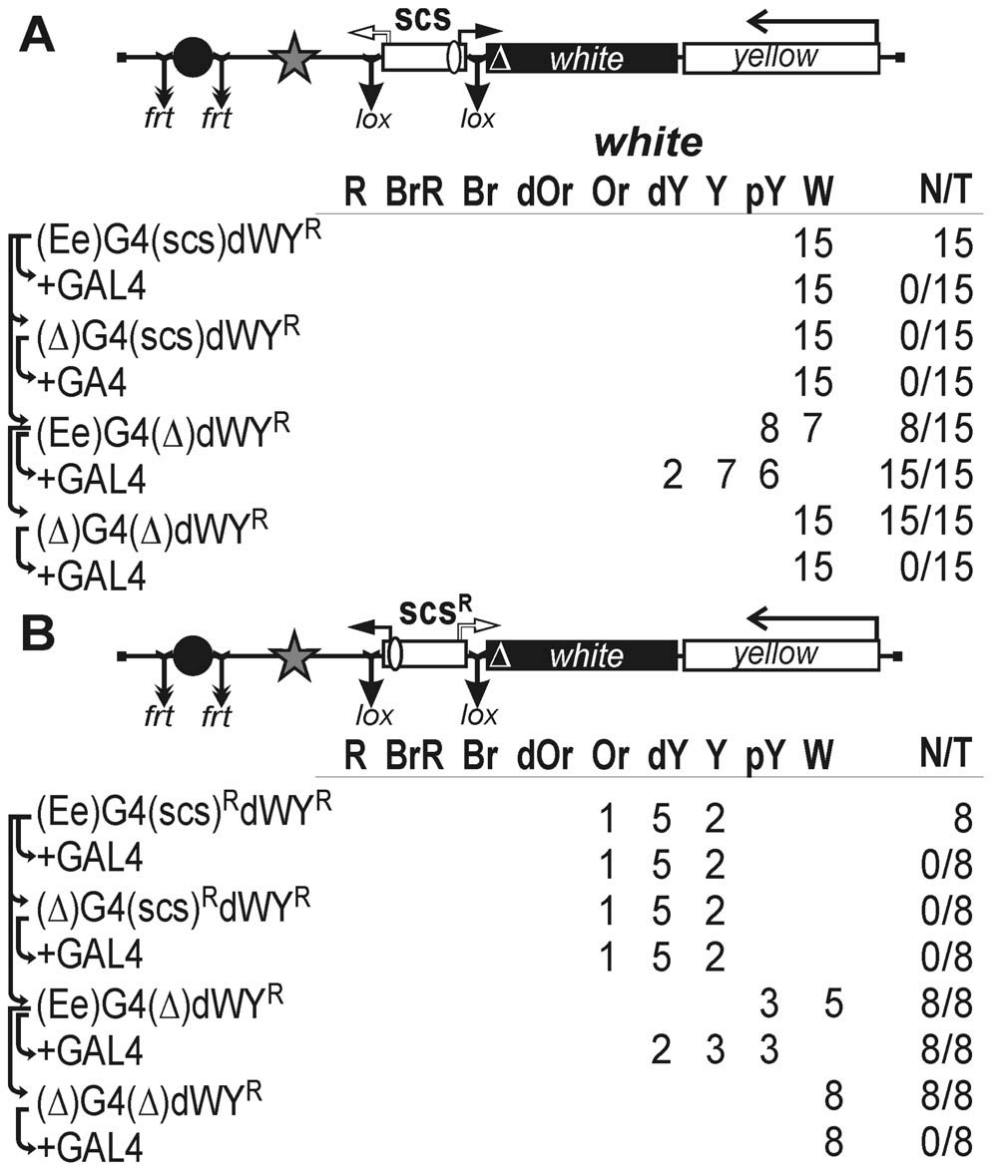
Testing (A) *CG31211* or (B) *Cad87A* promoter in the scs insulator. Index “R” indicates that the scs is inserted in the reverse orientation. Delta sign (Δ) indicates deletion of the *white* promoter. The star indicates four GAL4 binding sites inserted near the eye enhancer. “+GAL4” indicates that eye phenotypes were examined in transgenic lines after induction of GAL4 expression. Other designations are as in Figures 1 and 2.

To find out if the promoters of scs are active in the eyes, we inserted the lox-flanked scs insulator upstream of the promoterless *white* gene in either direct (Figure 6A) or reverse orientation (Figure 6B). The eye enhancer flanked by frt sites and five GAL4-binding sites was inserted upstream of the scs insulator. As shown previously [53], the eye enhancer can substitute the promoter and drive transcription of the *white* gene in the eyes.

We obtained 15 transgenic lines carrying the scs insulator inserted in direct orientation (Figure 6A). Flies of all these lines had white eye color, indicating that part of the *CG31211* promoter included in scs was inactive in the eyes. Induction of GAL4 produced no change in eye pigmentation. When the scs insulator was deleted, flies with pale yellow eyes appeared in half of transgenic lines. Moreover, eye pigmentation further increased after GAL4 induction, suggesting that GAL4 stimulated transcription from the eye enhancer. The lack of *white* expression in the presence of the scs insulator could be explained by its function as a terminator of transcription initiated at the eye enhancer.

In eight transgenic lines carrying the construct with the scs insulator inserted in reverse orientation, flies had eye pigmentation ranging from yellow to orange (Figure 6B). Deletion of the scs insulator significantly reduced eye pigmentation, suggesting the main role for the *Cad87A* promoter in *white* expression. Induction of GAL4 or deletion of the eye enhancer had no effect on eye pigmentation, indicating that the eye enhancer failed to stimulate the *Cad87A* promoter. However, the *Cad87A* promoter could affect the activity of the eye enhancer by transcription interference in transgenic lines described in Figure 6A.

**Figure 7:**
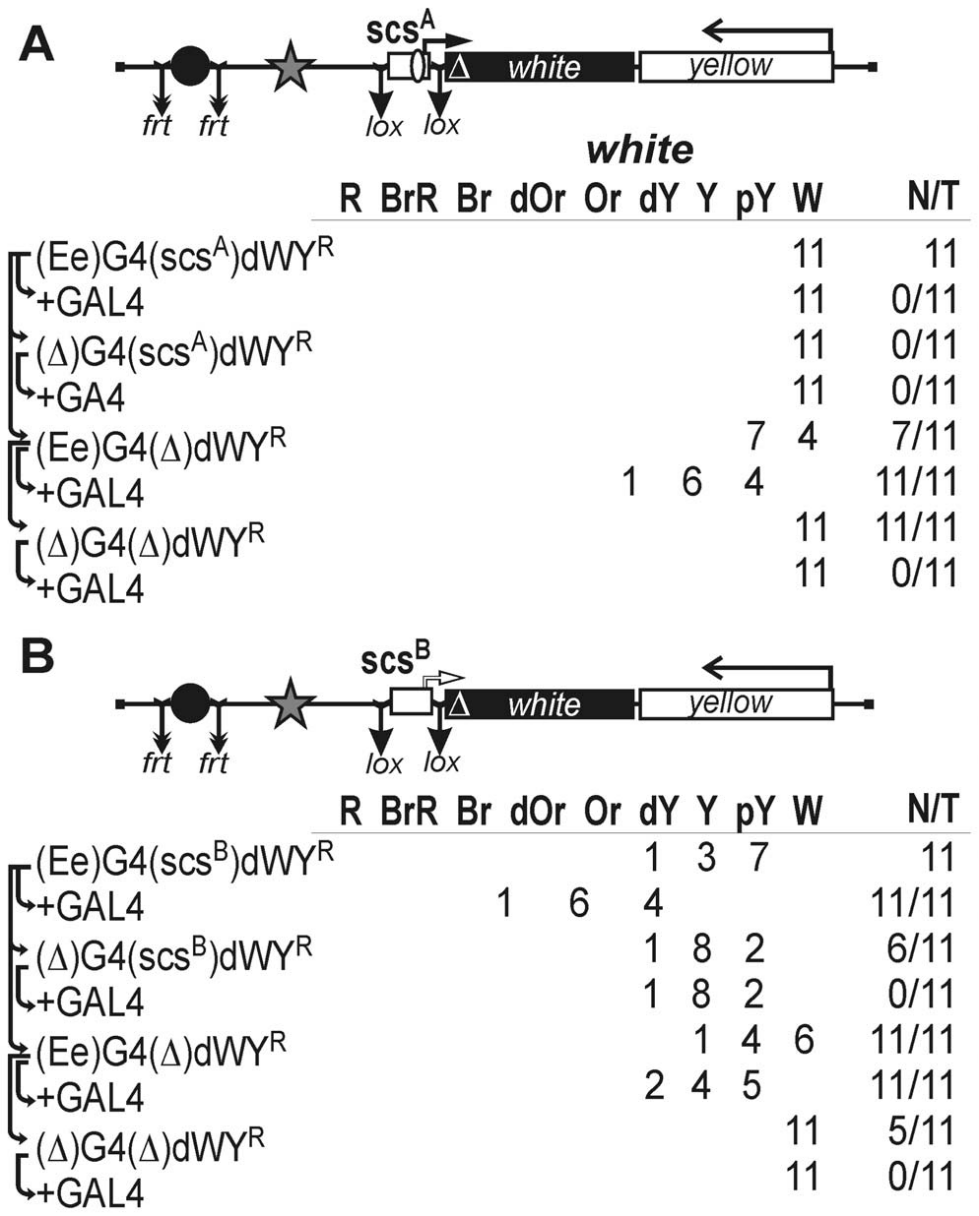
Testing (A) *CG31211* or (B) *Cad87A* promoter in the scs insulator. Other designations are as in Figures 1, 2, and 5. The scs^A^ is 516-bp scs part including the *CG31211* promoter. The scs^B^ is 477-bp scs part including the *Cad87A* promoter. Other designations are as in Figures 1, 2 and 5.

These results suggested that the eye enhancer failed to stimulate promoters contained in scs. However, it was possible that a certain region of the whole element blocked the interaction of the eye enhancer with the scs promoter. To test such a possibility, we inserted the lox-flanked parts of scs, including the *CG31211* promoter (516-bp scs^A^, Figure 7A) and the *Cad87A* promoter (477-bp scs^B^, Figure 7B), into the promoterless *white* gene.

Transgenic flies carrying scs^A^ had white eye color, indicating that the *CG31211* promoter was inactive in the eyes. Induction of the eye enhancer by GAL4 provided for an increase in eye pigmentation only after scs^A^ was deleted (Figure 7A), which was evidence for the role of the transcription terminator in interrupting transcription initiated from the eye enhancer.

Next, we examined 11 transgenic lines carrying the scs^B^ part of scs (Figure 7B). In all transgenic lines, flies had pigmented eyes, indicating the ability of the *Cad87A* promoter to drive the *white* expression in eyes. Deletion of the eye enhancer reduced eye pigmentation in most of transgenic lines, which might be explained either by the ability of the eye enhancer to weakly stimulate the *Cad87A* promoter or by the additive effect of transcription from the eye enhancer and the promoter. In any case, neither GAL4 activator nor the eye enhancer could effectively stimulate the *Cad87A* promoter.

In our previous study, transcription through the *mini-white* gene was found to result in a high level of its expression (from orange to red) in 38 (25%) out of 154 transgenic lines tested [26]. Deletion of the *white* promoter in these lines had no effect on eye pigmentation because of transgene insertion into the transcribed regions of genes that were active in the eye imaginal disks. Here, 23 derivative transgenic lines were obtained after deletion of the scs and the eye enhancer, and flies in all these lines had white eyes (Figure 6). In all 22 transgenic lines carrying scs^A^ and scs^B^ (Figure 7), the transgene was also inserted into genome regions that failed to support expression of promoterless *mini-white* gene. Thus, the transgenes carrying the scs insulator are rarely inserted into the genes expressed in the eye imaginal discs.

### Testing the scs' Insulator in the promoterless *white* Assay

**Figure 8:**
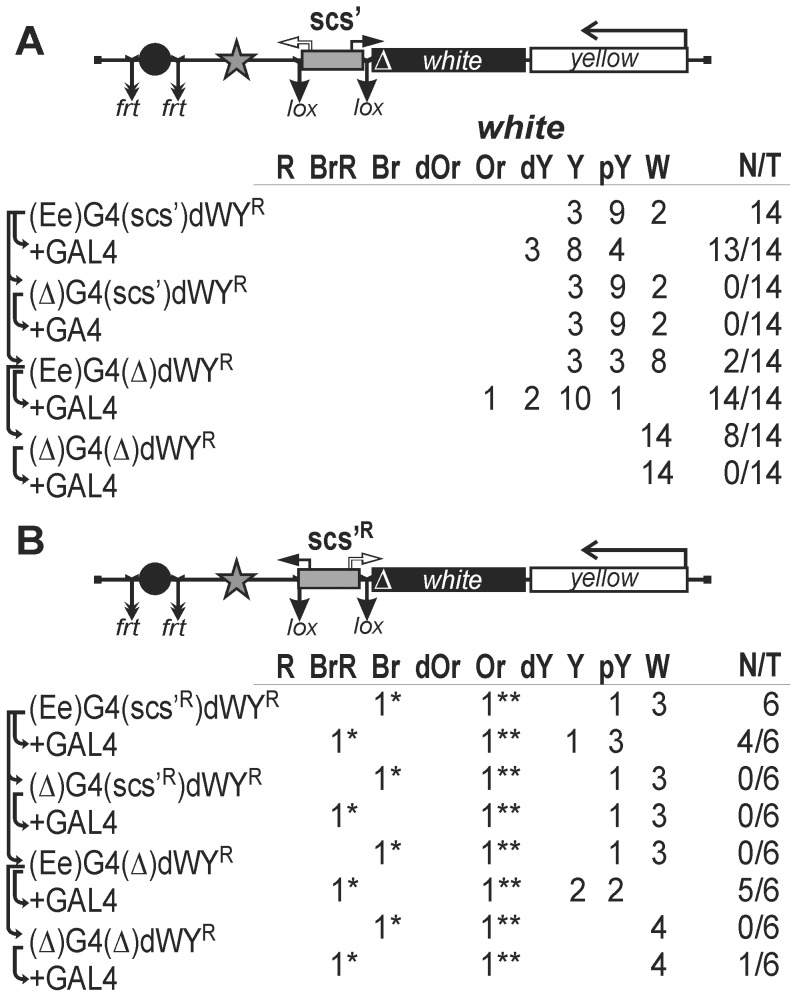
Testing (A) *aurora* or (B) *CG3281* promoter in the scs' insulator. Asterisks indicate that the transgene was codirectionally inserted (*) in the first intron of the *effet* gene (3R:10565091) transcribed in the same direction as the *mini-white* gene or (**) in the first intron of the *Dek* gene (2R:12744143). Other designations are as in Figures 1, 2 and 5.

According to the results obtained in S2 cells, the scs' insulator contains two functional promoters. To determine the activity of these promoters in eye imaginal disks, we inserted the scs' insulator flanked by lox sites into the promoterless *mini-white* gene in either direct (Figure 8A) or reverse orientation (Figure 8B). The eye enhancer flanked by frt sites and five GAL4 binding sites were inserted upstream of the scs' insulator. Deletion of the eye enhancer in the transgenic lines carrying the scs' insulator inserted in the direct orientation (Figure 8A) did not change eye pigmentation, indicating that the *aur* promoter was functional in the eye imaginal disks. In contrast, the *CG3281* promoter failed to drive *white* transcription (Figure 8B). Irrespective of scs' orientation, deletion of the eye enhancer had no significant influence on *white* expression, suggesting that the promoters in scs' are not sensitive to the *white* enhancer. GAL4 could weakly stimulate *white* expression only in the presence of the eye enhancer. This result confirms that the scs' insulator does not terminate transcription initiated in the eye enhancer.

In two transgenic lines, flies had strongly pigmented eyes, with the pigmentation level remaining unchanged after deletion of the scs' insulator and the eye enhancer. The localization of insertion sites in these transgenic lines showed that the transgene was inserted into the genes whose transcription direction coincided with that of the *mini-white* gene (Figure 8B). Thus, the scs' insulator fails to protect the *mini-white* gene from transcription starting upstream of the transgene integration site.

### Testing the scs and scs' Insulators in Enhancer-blocking and CPE Assays

**Figure 9:**
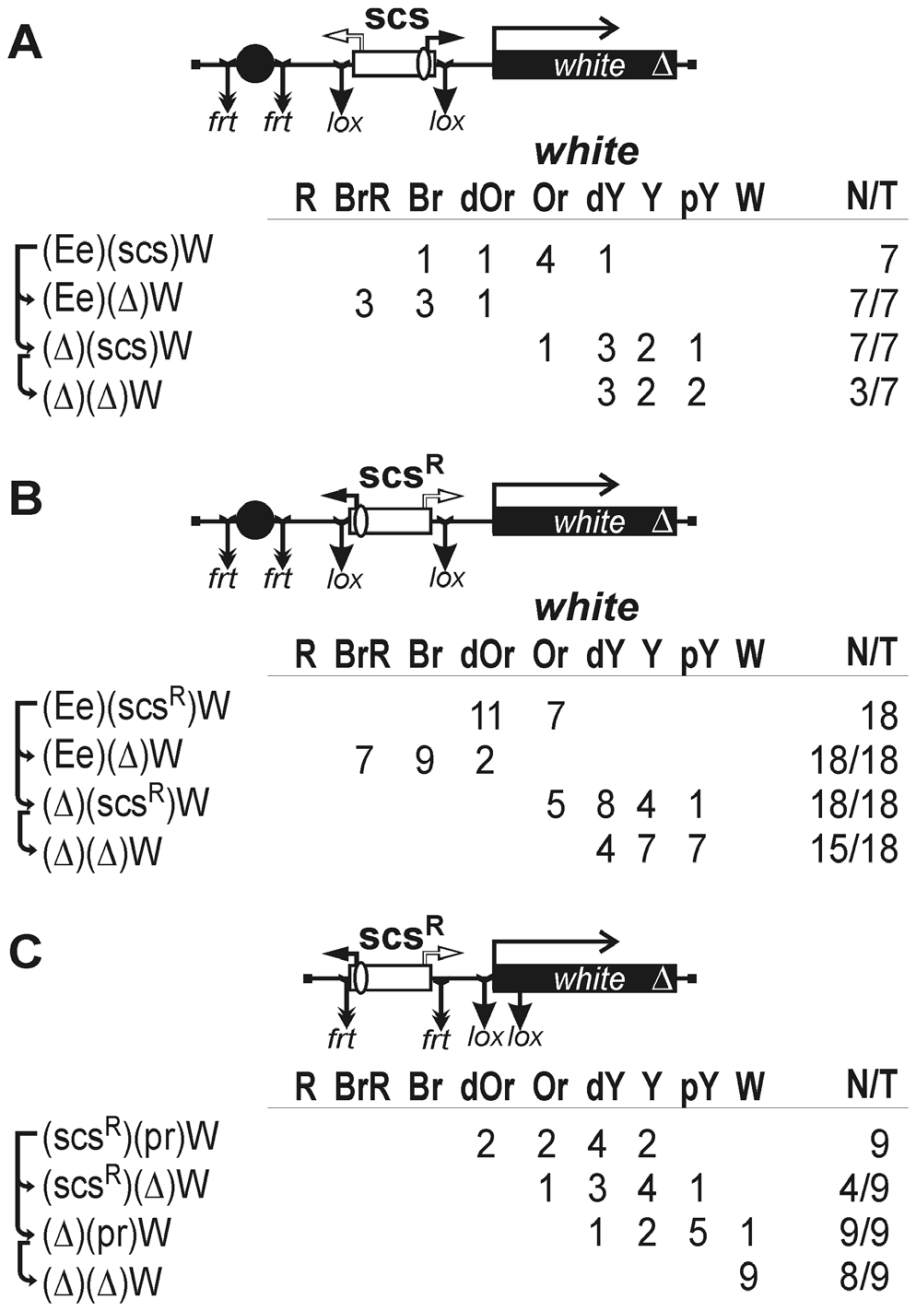
Testing the scs insulator inserted in (A) direct or (B) reverse orientation in enhancer-blocking and CPE assays and (C) testing for interference between the *white* promoter and the *Cad87A* promoter in the scs insulator. Other designations are as in Figures 1 and 2.

In the transgenic lines described in Figures 2 and 3, the eye enhancer was partially attenuated by the *yellow* promoter. To check whether the scs insulator could block the strong enhancer–promoter communication, we used the eye enhancer–*white* gene system with the deleted Wari insulator. The scs insulator flanked by lox sites was inserted in either direct (Figure 9A) or reverse orientation (Figure 9B) between the frt-flanked eye enhancer and the *white* promoter. In the resultant 25 transgenic lines, the scs insulator only partially blocked the eye enhancer activity. This was confirmed by the fact that deletion of the eye enhancer resulted in further reduction of eye pigmentation: in all 25 derivative transgenic lines with the deleted eye enhancer, flies had eye color phenotypes ranging from pale yellow to orange. Additional deletion of scs in any of the lines did not provide for an increase in eye pigmentation. This observation confirms our finding that the scs insulator directs integration of the transgenic construct into the genome regions that do not stimulate *mini-white* expression.

We noticed that eye pigmentation was darker in flies from transgenic lines carrying the scs insulator inserted in reverse orientation (Figures 9A, 9B), which could be explained by the activity of the *Cad87A* promoter. To test for cooperation between the *white* and *Cad87A* promoters, we made the construct in which the frt-flanked scs was inserted in reverse orientation upstream of the lox-flanked *white* promoter (Figure 9C). Transgenic flies had eye color phenotypes in the range from dark orange to yellow, and deletion of either scs or the *white* promoter reduced eye pigmentation, suggesting that the *Cad87A* and *white* promoters cooperate in the *mini-white* gene expression.

**Figure 10:**
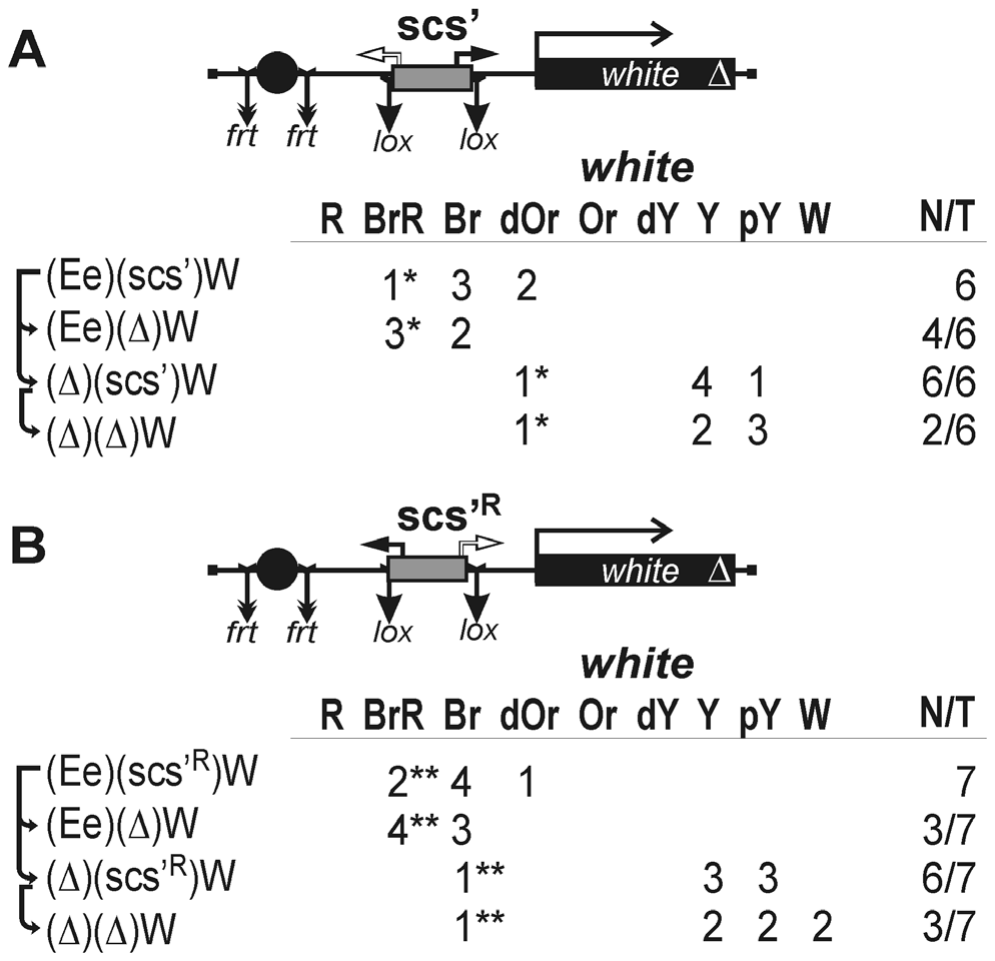
Testing the scs' insulator inserted in (A) direct or (B) reverse orientation in enhancer-blocking and CPE assays. Asterisks indicate that the transgene was codirectionally inserted (*) in an intron of the *capr* gene (3L:18665421) or (**) in an intron of the *CG7950* gene (3R:25882796). Other designations are as in Figures 1 and 2.

Next, we tested the scs' insulator in the enhancer blocking assay (Figure 10). We found that scs' failed to effectively block the eye enhancer: deletion of scs' led to a slight enhancement of eye pigmentation in only 7 out of 13 transgenic lines. At the same time, flies from two transgenic lines had a relatively high level of eye pigmentation after deletion of scs' and the eye enhancer. In both these lines, the transgene was inserted into genes whose transcription direction coincided with that of the *mini-white* gene (Figure 10). These results support our conclusion that scs' does not protect *white* expression from transcription initiated upstream of the transgene.

## Discussion

Our experiments with transgenic lines support previous observations [28], [48]–[51], [56] that scs is one of the strongest *Drosophila* insulators, while scs' has only a weak insulator activity. Here, we provide the first experimental evidence that pairing between scs and scs', described previously [33], has a functional outcome in improving the scs' insulator activity. The scs and scs' insulators in transgenic lines were located at a distance of about 9 kb, which is similar to the 14-kb distance between them in the endogenous genome region. As expected, scs is much more effective in reinforcing insulation mediated by Zw5 binding sites, which supports our previous observation that Zw5 can facilitate long-distance interactions [42]. The improvement of enhancer blocking by the interaction of scs and scs' may be explained by the formation of a chromatin loop between these insulators, which interferes with enhancer–promoter communication. However, it is also possible that the interaction between these insulators facilitates cooperative binding of insulator proteins to their sites, with consequent reinforcement of their enhancer-blocking activity.

The scs and scs' insulators contain promoters that are active in S2 cells, suggesting that both insulators may block enhancers according to the promoter competition model [40]. However, only the *Cad87A* promoter of scs and the *aur* promoter of scs' can drive *white* expression in the eyes. Since the *CG31211* gene is strongly transcribed in eyes (FlyBase database), we suggest that the scs insulator lacks certain regulatory elements that are important for the activity of this promoter. Unexpectedly, we have found that the eye enhancer or GAL4 fails to effectively stimulate the *Cad87A* and *aur* promoters. This may be explained either by the specificity of the eye enhancer to stimulate only the *white* promoter or by the inability of the promoters in scs and scs' to be stimulated by the activators bound to the enhancer or GAL4.

In transgenic lines, the scs insulator located on the 3' side of the *yellow* gene interacts with the promoter [54]. The scs insulator blocks the enhancers to the same extent in all transgenic lines, indicating that chromatin loop formation with insulators located outside the transgene is not required for enhancer blocking. Our results are in accordance with the previous observation that one copy of the scs insulator can block the eye enhancer on an episome, out of the chromatin context [56]. Taken together, these observations suggest that direct interactions of proteins bound to the scs modules and the *white* enhancer and/or promoter are responsible for effective blocking of the eye enhancer. Interestingly, even eight binding sites for the Zw5 protein block the enhancers to a much lesser extent than does the scs insulator that has only one Zw5 binding site [31]. This is evidence that additional, as yet unidentified proteins are required for insulation mediated by scs.

As shown previously, transcription induced by the *Cad87A* promoter of scs inserted into the regulatory region of the *bithorax* complex can affect the activity of the enhancers that stimulate *Abd-a* and *Abd-B* genes [35]. In contrast to our previous observation that transcription through the transgene inactivates the *mini-white* promoter [26], it has been found that transcription from the *Cad87A* promoter does not interfere with activity of the *white* promoter. When the *white* and *Cad87A* promoters have the same direction in transgenic lines, they function additively in stimulating *white* expression.

A number of experiments performed to date indicate that a major portion of the genome is being transcribed and that a large percentage of the transcripts is accounted for by long non-protein-coding sequences (lncRNA) [57]; [58]. Recent data suggests that many of lncRNA have important roles in regulation of transcription [59]. Therefore, to functionally separate two adjacent chromatin domains, the boundaries should contain transcription terminators. Here, we have found that the scs insulator contains terminators that stop transcription. Interestingly, SF1, a chromatin boundary located in the *Drosophila Antennapedia* complex (ANT-C) [60], also contains a functional transcription terminator (DL and OM, unpublished). Thus, the presence of transcription terminators may well be a common feature of chromatin boundaries.

In the transgenic assay used to test insulators for protection from chromosomal position effects (CPE), transcription terminators contained in the insulators could be partially responsible for CPE suppression. For example, we have shown previously that the SV40 transcriptional terminator was efficient in protecting *mini-white* expression from positive position effects [26]. In 4 out of the total 33 transgenic lines carrying the construct with scs' (12%), transcription through the transgene led to *white* expression in eyes. The scs' insulator failed to terminate such transcription, indicating that this insulator could not effectively protect from CPE. In addition to the ability of scs to terminate transcription, we found that this insulator reduced the frequency of insertion of the transgene into the regions that stimulate *white* expression in the eyes. It is possible that proteins bound to the scs insulator interact with chromatin proteins that recruit the transgene to certain genomic regions. Our results contradict the data by Cuvier et al. [21] who obtained flies with strongly pigmented eyes in 6 out of 19 transgenic lines (32%) carrying the *mini-white* gene flanked on the 3' side by the scs insulator. To explain the difference, we hypothesize that the *white* promoter and the Wari insulator also determine the sites of transgene insertion. We have previously found that the Wari insulator interacts with the *white* promoter and potentiates its activity [54]. In the experiments by Cuvier et al. [21], the transgene contained both regulatory elements of the *white* gene, while in our constructs either the promoter or insulator was deleted and, consequently, only the scs insulator was involved in determining the transgene insertion site. It is noteworthy that, as shown previously, human matrix attachment regions (MARs) can insulate transgene expression from CPE in *Drosophila melanogaster* [22]. However, excision of MARs from the transgenes has proved to have no effect on *white* expression [23]. It has been suggested that MAR sequences can cause transposon to home to the nuclear matrix prior to integration, thereby targeting transposon integration to specific kinds of sites within the *Drosophila* genome. Such genome region-specific targeting of transgenes carrying regulatory elements such as scs and MARs may reflect global organization of the genome [38], [61], [62]. Further study is required to understand the mechanisms of this phenomenon.
